# A recombinant Newcastle disease virus-vectored multi-epitope vaccine confers protection against QX-like infectious bronchitis virus in chickens

**DOI:** 10.3389/fimmu.2026.1949497

**Published:** 2026-09-22

**Authors:** Fei Wang, Jianan Xie, Guoyuan Wen, Xusheng Qiu, Yang Qu, Yingjie Sun, Cuiping Song, Ying Liao, Chan Ding, Shunlin Hu, Lei Tan

**Affiliations:** 1 Shanghai Veterinary Research Institute, Chinese Academy of Agricultural Sciences, Shanghai, China; 2 Institute of Animal Science and Veterinary Medicine, Hubei Academy of Agricultural Sciences, Wuhan, Hubei, China; 3 School of Agriculture and Biology, Shanghai Jiao Tong University, Shanghai Key Laboratory of Veterinary Biotechnology, Key Laboratory of Urban Agriculture (South), Ministry of Agriculture, Shanghai, China; 4 College of Veterinary Medicine, Yangzhou University, Yangzhou, China

**Keywords:** immune-protection, infectious bronchitis virus, multi-epitope vaccine, Newcastle disease virus, QX-like genotype, vaccine vector

## Abstract

Infectious bronchitis virus (IBV) QX-like (GI-19) genotype dominates global poultry flocks, yet Mass-type vaccines fail to confer cross-protection. We developed a recombinant Newcastle disease virus (NDV) LaSota vector expressing conserved IBV S1 B-cell hotspots paired with S1-derived BF2-restricted CTL epitopes (rNDV-IBV-T/B). Specific-pathogen-free chickens received single or booster ocular-nasal immunization (10^6^ EID_50_) followed by QX-like challenge. Booster immunization increased the frequency of splenic CD3^+^CD8^+^ T cells (41.3% vs. 31.9% in the sham-vaccinated challenge control; Tukey-adjusted p<0.001) and elicited neutralizing antibodies (peak log_2_ GMT, 5.80). Vaccinated groups showed markedly reduced clinical scores, preserved tracheal ciliary activity at all assessed post-challenge time points, and mitigated histopathological lesions. Vaccination reduced oropharyngeal and cloacal viral RNA shedding at multiple post-challenge time points, with the booster regimen producing a more consistent reduction in cloacal viral RNA. Protection was accompanied by an increased frequency of splenic CD3^+^CD8^+^ T cells, particularly after booster immunization, suggesting a role for cellular immunity alongside neutralizing antibodies. The NDV-LaSota backbone functions as a modular platform for rapid vaccine redesign against emerging coronavirus variants.

## 1 Introduction

Newcastle disease (ND) and infectious bronchitis (IB) are among the most economically consequential viral diseases of poultry worldwide, frequently co-circulating in intensive production systems and causing compounded losses through respiratory disease, nephropathogenesis, reduced egg production, and secondary bacterial infections (1, 2). ND is caused by Newcastle disease virus (NDV, Avulavirus, Paramyxoviridae), for which live attenuated LaSota-type vaccines confer robust protection but interfere with concurrently administered IBV vaccines (3, 4). IB is caused by infectious bronchitis virus (IBV), a Gammacoronavirus whose spike (S) protein—post-translationally cleaved into hypervariable S1 (receptor-binding, neutralizing epitopes) and conserved S2 (fusion)—drives both serotype definition and vaccine escape (5–8).

IBV is classified into nine major genotypes (GI–GIX) based on full S1 phylogeny, with GI further subdivided into >30 sublineages that largely map to serotypes and vaccine backbones (9, 10). Globally, Mass-type (GI-1, e.g. H120, M41) and 4/91-type (GI-13) remain the dominant vaccine backbones, while field prevalence has shifted toward QX-like (GI-19) in East Asia and Europe, TW-like (GI-7) in Southeast Asia, and, recently, GVI-1 in southern China (11, 12). In China, GI-19 (QX) has prevailed since the late 2000s, accounting for >70% of field isolates in eastern provinces, with sporadic incursions of GI-13 (4/91) and emergent GVI-1 that escape current H120 + LaSota combinations (13). The S1 subunit evolves at ~3% per year; its three hypervariable regions (HVRs) and major conformational neutralizing epitopes diverge strongly between Mass and QX (S1 nucleotide identity <80%), such that Mass-based live vaccines afford limited cross-protection against QX, contributing to endemic “vaccination failures” despite high coverage (14–16).

Conventional control relies on monovalent NDV (LaSota) and IBV (H120, QXL87, 4/91) live vaccines, but coadministration suffers from viral interference, and single-antigen IBV vaccines poorly cover heterologous genotypes (17–19). Recombinant NDV-vectored vaccines expressing IBV antigens have emerged as a bivalent strategy to bypass interference while broadening IBV coverage (20, 21). Early constructs expressed full-length IBV S1 or S under NDV LaSota, which protected against homologous M41 but gave inconsistent cross-protection against QX-like strains unless paired with a booster (22–24). An alternative is the multi-epitope approach, which focuses the immune response on conserved neutralizing B-cell hotspots and CTL epitopes while minimizing vector payload. Our group previously mapped three conserved S1 neutralizing B-cell epitopes (24–61, 132–149, 291–398) and BF2-restricted CTL epitopes (P8, P9 derived from QX-like SH1208) from the S1 subunit (25, 26). These were engineered into a recombinant NDV LaSota backbone (rNDV-IBV-T/B, where “rNDV” denotes recombinant NDV-LaSota) that protected SPF chickens against prototype M41 challenge (27). Independently, Qin et al. constructed a related NDV-LaSota-vectored multi-epitope vaccine (rLaSota/SBNT) incorporating four N-protein T-cell epitopes and the same three S1 B-cell hotspots, which conferred cross-protection against QX-like SD and TW-like GD strains but failed against M41—attributed to the N-CTL repertoire being insufficient when B-cell epitope conservation alone could not bridge the M41–QX serotype gap (28).

Notably, whether rNDV-IBV-T/B—which shares the NDV-LaSota backbone with rLa Sota/SBNT but carries S1-derived CTL rather than N-derived CTL, and was only tested against M41—can protect against a genuine QX-like challenge remains undefined (27). This question is directly relevant to field application in China, where QX has dominated for over a decade. Additional conserved B-cell targets have also been identified in the S2 HR2 region, highlighting the potential value of epitope-focused strategies across divergent IBV lineages (29). Here, we comprehensively assessed rNDV-IBV-T/B immunogenicity and efficacy against a QX-like challenge in SPF chickens, comparing single-dose vs. booster ocular-nasal immunization. We measured CD3^+^CD8^+^ T-cell expansion, QX-specific neutralizing antibodies, tracheal ciliostasis, histopathological examination of tracheal and renal tissues, and serial cross-sectional assessment of oropharyngeal and cloacal viral RNA loads over 14 days post-challenge (dpc). The results demonstrate that rNDV-IBV-T/B confers effective protection against QX, with the booster regimen producing a more consistent reduction in cloacal viral RNA loads—supporting its development as a bivalent ND–IB candidate for QX-endemic regions.

## 2 Materials and methods

### 2.1 Animals, viruses, vaccines, cells, and reagents

Specific-pathogen-free (SPF) embryonated chicken eggs were purchased from Zhejiang Lihua Agricultural Technology Co., Ltd. One-day-old SPF White Leghorn chickens were obtained from Boehringer Ingelheim Vital Biotechnology Co., Ltd. (Jiangsu, China) and housed in negative-pressure isolators with ad libitum access to sterile water and feed. The recombinant vaccine strain rNDV-IBV-T/B was rescued from our previously established NDV LaSota reverse genetics system as described (27). The QX-type IBV challenge strain was obtained from the China Institute of Veterinary Drug Control (Beijing, China) and stored at -80 °C. The 50% embryo infectious dose (EID_50_) of both viruses was calculated by the Reed-Muench method (30). HeLa cells (ATCC CCL-2) were maintained in our laboratory and cultured in Dulbecco’s Modified Eagle Medium (DMEM; Gibco) supplemented with 10% fetal bovine serum (FBS; ExCell Bio) and 1% penicillin-streptomycin (Beyotime). Mouse Anti-Chicken CD4-PE, Mouse Anti-Chicken CD8α-FITC, Mouse Anti-Chicken CD3-APC (SouthernBiotech); Chicken Tissue Lymphocyte Isolation Kit (Haoyang Biotechnology); SYBR Green qPCR Mix (Vazyme Biotech Co., Ltd.).

### 2.2 Immunofluorescence verification of exogenous protein expression

As previously described (27) with the following modifications: HeLa cells were seeded in 6-well plates and infected at a multiplicity of infection (MOI) of 1 with rNDV-IBV-T/B, parental LaSota, or mock-infected with PBS. At 24 h post-infection, cells were fixed with 4% paraformaldehyde, permeabilized with 0.5% Triton X-100, and blocked with 3% BSA. Serum from SPF chickens that had survived QX strain challenge (1:200 dilution) was used as the primary antibody, and FITC-conjugated goat anti-chicken IgY (1:500) as the secondary antibody. Nuclei were counterstained with DAPI. Fluorescence was visualized using a Zeiss LSM 800 laser confocal microscope.

### 2.3 Evaluation of the protective efficacy of rNDV-IBV-T/B

Sixty SPF chickens were randomly allocated using a computer-generated random-number sequence to three experimental groups (n=20 per group): Group A, sham-vaccinated challenge control; Group B, single-dose rNDV-IBV-T/B; and Group C, booster-dose rNDV-IBV-T/B. An additional five unvaccinated and unchallenged chickens were maintained separately as naïve baseline controls.

Single-immunization group: Received one dose of rNDV-IBV-T/B (10^6^ EID_50_ in 0.2 mL) via ocular-nasal route at 3 days of age; Booster-immunization group: Received the same primary dose at 3 days of age, followed by a second identical dose at 24 days of age (14 days before challenge); Control group: Received 0.2 mL PBS via the same route at 3 days of age. At 14 days post-booster immunization (38 days of age), the 17 remaining chickens in each experimental group were challenged with 0.2 mL of QX-type IBV (10^6^ EID_50_) via ocular-nasal route. Chickens were observed daily for 14 days post-challenge (dpc) for clinical signs by an observer blinded to group assignment. Clinical scores were assigned as previously described (27): 0 = normal; 1 = one symptom category; 2 = two symptom categories; 3 = all three symptom categories; 4 = death.

### 2.4 Evaluation of cellular immunity

At 14 days post-booster immunization (immediately prior to challenge), 3 chickens per group were randomly selected for euthanasia. Splenic lymphocytes were isolated using the Chicken Tissue Lymphocyte Isolation Kit per manufacturer instructions. Lymphocytes were adjusted to 5×10^5^ cells/mL and seeded in 12-well plates. For flow cytometric analysis, cells were stained with APC-CD3, PE-CD4, and FITC-CD8α antibodies (0.5 μL per well) and incubated at 4 °C in the dark for 30 min, followed by two washes with PBS containing 2% FBS. The fluorochrome-conjugated antibodies were selected based on the manufacturer-documented reactivity with chicken antigens. Single-stain controls were included for each fluorochrome (APC-CD3, PE-CD4, and FITC-CD8α) to establish fluorescence-compensation settings and verify the separation of signals in the corresponding detection channels. Lymphocytes were identified using FSC-A versus SSC-A, followed by singlet discrimination using FSC-A versus FSC-H. Data were acquired on a NovoCyte flow cytometer and analyzed using FlowJo v10.0.

### 2.5 Embryonated chicken egg neutralization assay

Serum was collected from 5 randomly selected chickens per group at 14, 21, and 28 days post-first immunization (dpfi). Neutralizing antibody titers against QX strain were determined as previously described (26) with modifications: heat-inactivated serum was serially diluted 2-fold, mixed with an equal volume of QX strain containing 100 EID_50_/0.1 mL, and incubated at 25 °C for 1 h. The mixture was inoculated into 6 SPF embryonated eggs per dilution (0.2 mL/egg). Eggs were incubated at 37 °C for 144 h and examined daily for embryo lesions/death. Serum was serially diluted 2-fold from 1:2 to 1:128; limit of detection (LOD) was 2 log_2_ (1:4 dilution). Neutralization titers were expressed as the reciprocal of the highest serum dilution that prevented embryo death or typical IBV lesions, and geometric mean titers (GMT) with 95% confidence intervals were calculated for each group. Virus and normal serum controls were included in each run.

### 2.6 Tracheal ring ciliostasis assay

As described previously (27), experimental chickens were humanely euthanized under aseptic conditions via intravenous injection of sodium pentobarbital (80 mg/kg BW) following American Veterinary Medical Association (AVMA) euthanasia guidelines. Death was confirmed by the absence of pedal withdrawal reflex, respiratory arrest, and cardiac standstill prior to tissue collection. For serial cross-sectional ciliostasis assessment, 2 chickens per group were euthanized at 5 and 7 dpc, and 3 chickens per group at 9 dpc. An additional 10 chickens per group were maintained until 14 dpc for terminal tissue collection. From this terminal cohort, 3 chickens were randomly selected for histopathological analysis at 14 dpc. The mid-cervical trachea was aseptically collected from each bird after removing surrounding connective tissue, then cut into 0.5–1 mm rings (10 rings/chicken). Ciliary activity was observed under an inverted microscope and scored blindly by two independent observers using the following 0–4 scale: 0 = 100% ciliary beating, 4 = complete ciliostasis; 1 = ciliostasis in ≤25% of cilia, 2 = ciliostasis in 25%–50% of cilia, 3 = ciliostasis in 50%–75% of cilia. For each chicken, the scores of the 10 tracheal rings were averaged to obtain one bird-level ciliostasis score. The chicken, rather than the individual tracheal ring, was treated as the biological replicate, and groups were compared separately at 5, 7, and 9 dpc.

### 2.7 Histopathological analysis

At 14 dpc, three chickens per group were independently selected at random from the 10-bird terminal cohort for descriptive histopathological examination. The chickens were humanely euthanized via intravenous injection of sodium pentobarbital (80 mg/kg BW) following AVMA guidelines for tissue collection; death was confirmed by the absence of pedal withdrawal reflex, respiratory arrest, and cardiac standstill prior to tissue collection. Major organs including lung, kidney, trachea, and bursa of Fabricius were harvested. The samples were cut into small pieces and fixed in 10% neutral buffered formalin for 48 h. After fixation, the tissues were dehydrated through graded ethanol solutions of 70%, 80%, 90%, 95%, and 100%, cleared in xylene for 30 min, and embedded in paraffin. Subsequently, the paraffin-embedded tissues were sectioned into 3–5 μm slices using a microtome. The sections were floated on a warm water bath, transferred onto glass slides, and dried in an oven at 60 °C for 30–60 min. The sections were dewaxed in xylene, rehydrated through graded ethanol solutions of 100%, 95%, and 70%, and rinsed with tap water. The sections were then stained with hematoxylin, differentiated in 1% acid alcohol, rinsed, blued in 0.5% ammonia water, counterstained with eosin, and rinsed again. After staining, the sections were dehydrated through ascending ethanol concentrations, cleared in xylene, and mounted with coverslips while avoiding air bubbles.

### 2.8 Detection of viral shedding post IBV QX strain challenge

At each sampling time point (2, 4, 6, 8, 10, 12, and 14 dpc), three chickens per group were selected at random from the cohort of 10 birds maintained until 14 dpc for terminal tissue collection, and paired oropharyngeal and cloacal swabs were collected. Selection was performed independently at each time point; therefore, the same chickens were not necessarily sampled repeatedly across all time points. The birds sampled for viral RNA detection thus represented time-specific random subsets of the 10-bird terminal cohort rather than a separate cohort. Swabs were placed in 1 mL sterile PBS containing antibiotics, vortexed, and stored at -80 °C until processing. Viral RNA was extracted using TRIzol reagent, and IBV loads were quantified by qRT-PCR using a previously established assay and minor modifications (27). Briefly, the IBV RNA was amplified using the forward primer IBV5’GU391 5’-GCTTTTGAGCCTAGCGTT-3’; the reverse primer IBV5’GL533 5’-GCCATGTTGTCACTGTCTATTG-3’, amplifying a 143-bp fragment of the IBV N gene, and detected with CFX96 real-time PCR detection system. Standard curves were generated from *in vitro*-transcribed IBV N gene RNA (10²–10^8^ copies/μL), with an amplification efficiency 98–102%, R² > 0.99 across all runs. Cycle threshold (Ct) values were automatically determined using Bio-Rad CFX Maestro software. Viral loads were expressed as log_10_ copies per swab.

### 2.9 Statistical analysis

For viral RNA-load analysis, the three technical qRT-PCR replicates from each chicken were averaged to obtain one value per biological replicate. At each time point, data are presented as the mean ± SD of three chickens per group. Measurements with detectable amplification but below the lower limit of quantification (LLOQ; 2 log_10_ copies/swab) were treated as left-censored observations and assigned one-half of the LLOQ on the original copy-number scale before log_10_ transformation. These observations were retained as biological replicates. Group differences were evaluated separately at each time point using one-way ANOVA followed by Tukey’s multiple-comparison test. All tests were two-sided, and the chicken was treated as the experimental unit. Given the small sample size (n=3 chickens per group at each time point), these comparisons were considered exploratory.

For the ciliostasis assay, the 10 tracheal-ring scores from each chicken were averaged to obtain one bird-level value. Group differences were analyzed separately at each time point using one-way ANOVA followed by Tukey’s multiple-comparison test. Data are presented as mean ± SD of bird-level values. Given the limited sample size (n=2 chickens per group at 5 and 7 dpc and n=3 per group at 9 dpc), these comparisons were considered exploratory. Clinical scoring, ciliostasis assessment, and histopathological evaluation were performed by observers blinded to group allocation.

## 3 Results

### 3.1 Immunofluorescence detection of T/B protein expression

Consistent with our previous report (27), rNDV-IBV-T/B successfully expressed the IBV multi-epitope cassette *in vitro*. Specific green fluorescence corresponding to the T/B protein was exclusively detected in HeLa cells infected with rNDV-IBV-T/B (**Figure 1A**), whereas no signal was observed in cells infected with parental LaSota (**Figure 1B**) or mock-infected controls (**Figure 1C**). Critically, the primary antibody used here was derived from chickens that had survived QX challenge, confirming that the epitopes expressed by the recombinant virus are recognized by antibodies elicited by circulating QX strains—a prerequisite for cross-protective humoral immunity.

**Figure 1:**
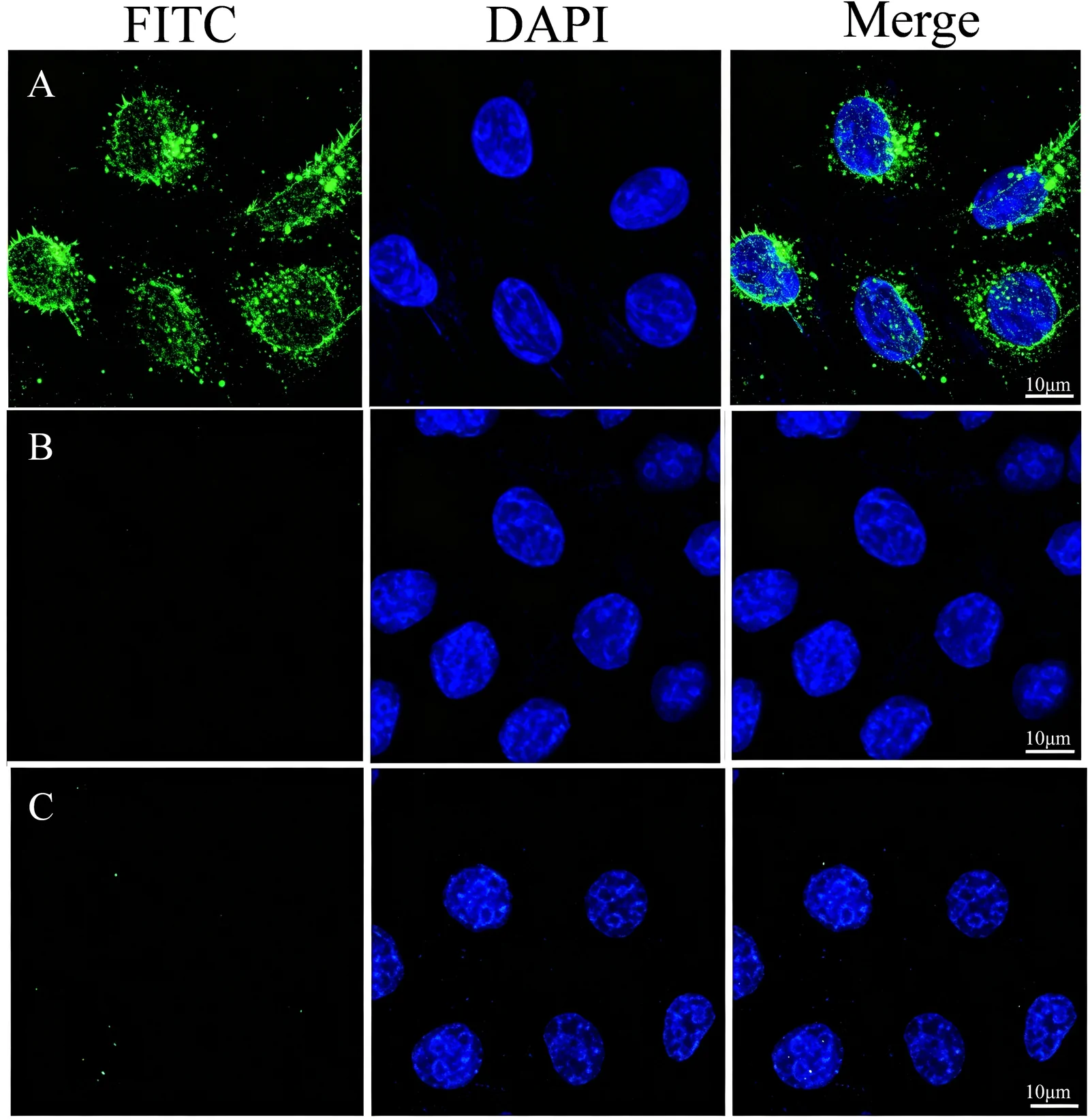
Immunofluorescence verification of IBV multi-epitope cassette expression in rNDV-IBV-T/B-infected cells. Representative confocal micrographs showing expression of the IBV T/B multi-epitope cassette in HeLa cells infected with rNDV-IBV-T/B **(A)**, parental LaSota strain **(B)**, or mock-infected with PBS **(C)**. Cells were fixed at 24 h post-infection, permeabilized, and blocked prior to incubation with primary antibody (1:200 dilution of serum from SPF chickens that survived QX-type IBV challenge) and FITC-conjugated goat anti-chicken IgY secondary antibody (1:500). Nuclei were counterstained with DAPI. Specific green fluorescence corresponding to the T/B cassette is exclusively detected in rNDV-IBV-T/B-infected cells, confirming detectable expression of immunogenic epitopes conserved in circulating QX strains. Images are representative of three independent infection experiments. Scale bars = 10 μm.

### 3.2 Evaluation of cellular immunity

Splenic T-cell subsets were analyzed by flow cytometry at 14 days post-booster immunization. Lymphocytes were first identified using FSC-A versus SSC-A to exclude debris, followed by singlet discrimination using FSC-A versus FSC-H. CD3^+^ T lymphocytes were subsequently gated, and CD8^+^ cytotoxic T cells and CD4^+^ helper T cells were resolved within the CD3^+^ population. **Figures 2A–C** show representative CD4/CD8 contour plots from three independent biological replicates per group, with Q1 representing CD3^+^CD8^+^ T cells and Q3 representing CD3^+^CD4^+^ T cells: Group A, sham-vaccinated challenge control; Group B, single-dose rNDV-IBV-T/B; and Group C, booster-dose rNDV-IBV-T/B. Quantitative analysis of CD3^+^CD8^+^ T-cell frequencies (**Figure 2D**) revealed a higher proportion in the booster-immunization group (Group C: 41.3% ± 0.6%) than in both the single-dose group (Group B: 38.4% ± 0.9%; Tukey-adjusted p = 0.0099) and the sham-vaccinated challenge control group (Group A: 31.9% ± 0.9%; p < 0.001). The single-dose group also showed a significantly higher CD3^+^CD8^+^ T-cell proportion than the sham-vaccinated challenge control group (p < 0.001). In contrast, CD3^+^CD4^+^ T-cell proportions remained comparable among Group A (24.2% ± 1.2%), Group B (22.4% ± 0.6%), and Group C (22.1% ± 1.5%), with no statistically significant overall difference (one-way ANOVA, p = 0.128). These findings show that vaccination, particularly the booster regimen, was associated with an increased frequency of splenic CD3^+^CD8^+^ T cells, whereas CD3^+^CD4^+^ T-cell frequencies remained similar among groups.

**Figure 2:**
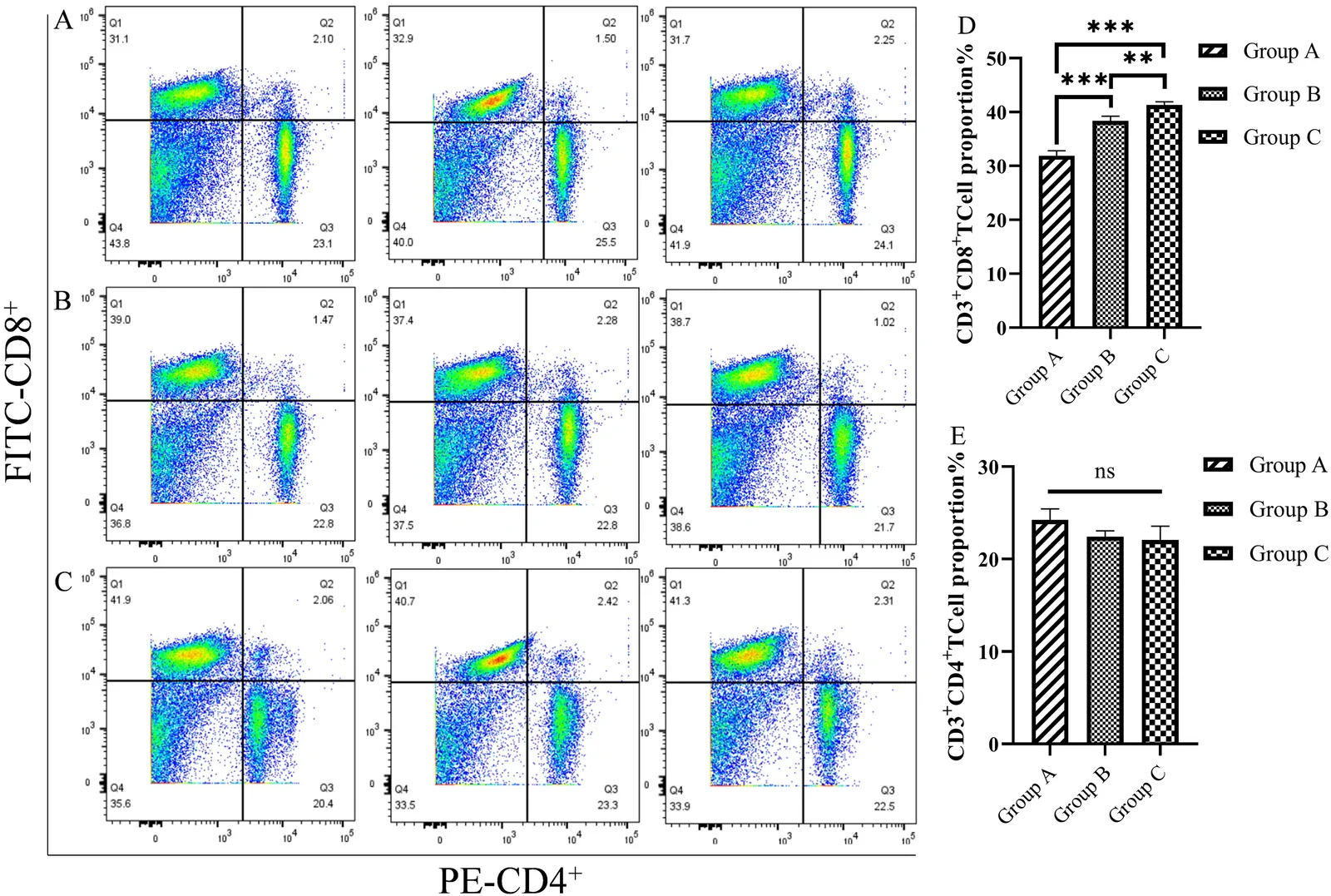
Flow cytometric analysis of splenic T cell subsets in immunized chickens. **(A–C)** Representative contour plots from three independent biological replicates per group. **(A)** Sham-vaccinated challenge control group (Group A), **(B)** Single-dose rNDV-IBV-T/B group (Group B), **(C)** Booster-dose rNDV-IBV-T/B group (Group C). CD3^+^ T cells were first gated from lymphocytes, and CD4^+^/CD8^+^ subsets were identified within the CD3^+^ gate (Q1 = CD3^+^CD8^+^, Q3 = CD3^+^CD4^+^). Single-stain controls for APC-CD3, PE-CD4, and FITC-CD8α were used to establish fluorescence-compensation settings before analysis of the experimental samples. **(D)** Quantitative bar graph of CD3^+^CD8^+^ T cell proportions. **(E)** Quantitative bar graph of CD3^+^CD4^+^ T cell proportions. Data are presented as mean ± SD (n=3 chickens per group). Overall group differences were assessed by one-way ANOVA, followed by Tukey-adjusted pairwise comparisons. For CD3^+^CD8^+^ T-cell frequencies, ***p < 0.001 for Group A versus Group B and Group A versus Group C, and **p < 0.01 for Group B versus Group C. The overall difference in CD3^+^CD4^+^ T-cell frequencies was not significant (ns). Samples acquired on a NovoCyte cytometer, analyzed with FlowJo v10.0.

### 3.3 Evaluation of humoral immunity

IBV QX-specific neutralizing antibodies were detectable in both immunized groups beginning at 14 days post-first immunization (dpfi) (**Table 1**), whereas all titers in the sham-vaccinated challenge control remained below the assay detection limit (<2 log_2_) at all examined time points. At 14 dpfi, the log_2_ GMT (95% CI) was 4.20 (3.64–4.76) in the booster-dose group and 3.40 (2.72–4.08) in the single-dose group; the between-group difference was not statistically significant after Holm adjustment (adjusted p=0.119). At 21 dpfi, the corresponding values were 5.80 (5.24–6.36) and 4.20 (3.64–4.76), respectively, with a significantly higher titer in the booster-dose group (p<0.05). At 28 dpfi, the booster-dose group maintained a log_2_ GMT of 5.20 (4.64–5.76), compared with 3.60 (2.92–4.28) in the single-dose group (p<0.05). Because all sham-control observations were below the detection limit and were treated as left-censored, no formal inferential comparisons with this group were performed. The peak QX-specific log_2_ GMT was numerically approximately 1.0 log_2_ lower than the value reported in our previous M41 study (27), yet sufficient to confer protection against QX challenge—supporting the hypothesis that the multi-epitope cassette targets conserved antigenic regions shared between M41 and QX.

**Table 1:** QX-specific neutralizing antibody titers [log_2_ GMT (95% CI)] in sera of immunized SPF chickens.

Group	14 dpfi	21 dpfi	28 dpfi
Sham-vaccinated challenge control	n.d. (<2)	n.d. (<2)	n.d. (<2)
Single-dose rNDV-IBV-T/B	3.40 (2.72–4.08)	4.20 (3.64–4.76)	3.60 (2.92–4.28)
Booster-dose rNDV-IBV-T/B	4.20 (3.64–4.76)	5.80 (5.24–6.36)#	5.20 (4.64–5.76)#

dpfi, days post-first immunization; GMT, geometric mean titer; CI, confidence interval; n.d., not detectable. Data are presented as log_2_ GMT (two-sided 95% CI) from five chickens per group. Confidence intervals were calculated on the log_2_ scale using Student’s t distribution (df=4). Because all values in the sham-vaccinated challenge control were below the assay detection limit (<2 log_2_), no confidence interval or formal inferential comparison with this group was performed. The booster-dose and single-dose groups were compared at each time point using two-sided exact permutation tests based on individual log_2_ titers, with Holm adjustment across the three time points. ^#^Holm-adjusted p<0.05 versus the single-dose group.

### 3.4 Clinical observation

Throughout the 14-day post-challenge period, no mortality was recorded in any group, and clinical scores remained low in vaccinated chickens (mean ± SD: single 0.8 ± 0.2, booster 0.4 ± 0.1) compared with the sham-vaccinated challenge control (2.9 ± 0.2, p<0.001 for both comparisons). The absence of QX-induced mortality herein contrasts with our previous report using the same rNDV-IBV-T/B backbone against M41, where sporadic deaths occurred in the M41-challenged control at 21 days of age (27). This discrepancy is primarily attributed to host age at challenge. In the present study, chickens were challenged at 38 days of age, an age at which resistance to QX-like (GI-19) infection is significantly higher; QX is classically nephropathogenic with high mortality in chicks <14 days of age, whereas mortality declines sharply in birds >28 days old even under experimental infection (14, 31). Conversely, the 2019 M41 trial challenged chickens at 21 days of age; although M41 is a respiratory-predominant genotype with historically low virulence in older birds, high-dose inoculation in younger SPF chickens can occasionally precipitate mortality due to severe respiratory distress and secondary complications (5, 15).

### 3.5 Tracheal ring ciliary activity after challenge

Ciliostasis scores were analyzed separately at 5, 7, and 9 dpc using the individual chicken as the experimental unit. Blind assessment revealed marked ciliostasis in the sham-vaccinated challenge control group (Group A). At 5 dpc, mean ciliostasis scores were 3.25 ± 0.07, 0.90 ± 0.14, and 0.30 ± 0.14 in Groups A, B, and C, respectively. The corresponding scores were 3.40 ± 0.28, 0.80 ± 0.28, and 0.45 ± 0.21 at 7 dpc, and 3.00 ± 0.00, 0.40 ± 0.10, and 0.17 ± 0.12 at 9 dpc. Both vaccinated groups exhibited significantly lower ciliostasis scores than Group A at all three time points. The booster-dose group also had lower scores than the single-dose group at 5 dpc (Tukey-adjusted p=0.0328) and 9 dpc (p=0.0405), whereas the difference between the two vaccination regimens was not significant at 7 dpc (p=0.469). These results indicate that both vaccination regimens preserved tracheal ciliary activity, with the booster regimen showing the lowest numerical ciliostasis scores across the assessed time points (**Figure 3**).

**Figure 3:**
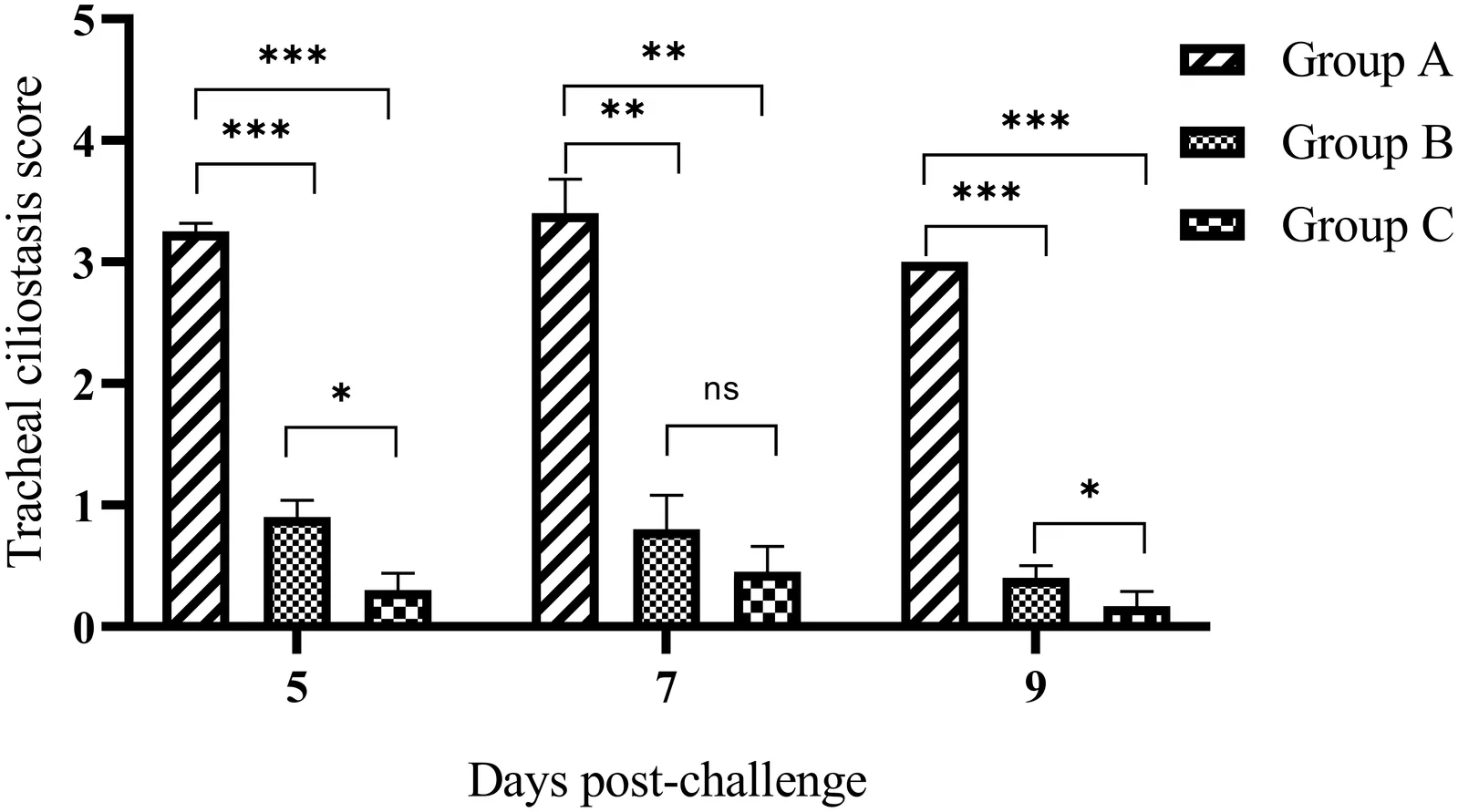
Tracheal ciliostasis scores in immunized chickens following QX-type IBV challenge. The bar graph shows bird-level mean ciliostasis scores at 5, 7, and 9 days post-challenge (dpc) using a serial cross-sectional design. Groups were defined as follows: Group A, sham-vaccinated challenge control; Group B, single-dose rNDV-IBV-T/B; and Group C, booster-dose rNDV-IBV-T/B. Ten tracheal rings were examined per chicken, with 2 chickens per group at 5 and 7 dpc and 3 chickens per group at 9 dpc. The 10 ring scores from each chicken were averaged to obtain one biological replicate. Bars and error bars represent the mean ± SD of bird-level values. Groups were compared separately at each time point using one-way ANOVA followed by Tukey’s multiple-comparison test. *p<0.05, **p<0.01, and ***p<0.001; ns, not significant.

### 3.6 Histopathological post IBV challenge

Histopathological examination of tracheal and kidney tissues at 14 dpc revealed qualitative findings that were consistent with the clinical, ciliostasis, and viral RNA-load results (**Figure 4**). In the trachea, the naïve baseline control group (**Figure 4A**) retained intact pseudostratified ciliated columnar epithelium with uniform ciliary alignment and no infiltrating inflammatory cells in the lamina propria. The single-dose and booster-dose groups showed qualitatively milder tracheal lesions, including more limited epithelial desquamation and less inflammatory-cell infiltration than the sham-vaccinated challenge control (**Figures 4B, C**). In stark contrast, the sham-vaccinated challenge control group (**Figure 4D**) displayed severe, diffuse epithelial denudation, extensive edema of the mucosa and submucosa, and dense mononuclear cell infiltration throughout the lamina propria, with near-total loss of ciliated epithelium.

**Figure 4:**
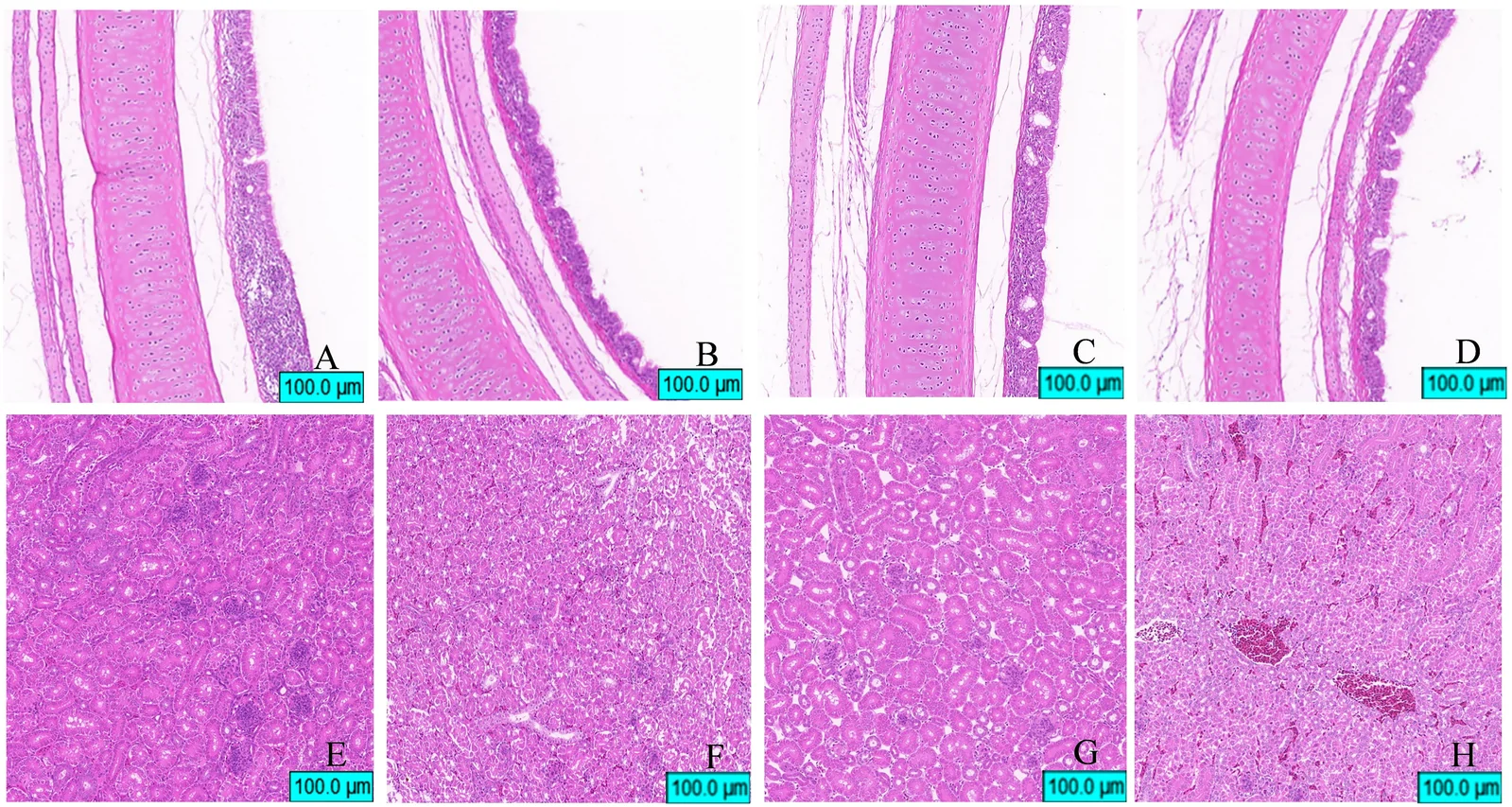
Histopathological analysis of tracheal and renal tissues post-QX-type challenge. Representative H&E-stained sections of trachea **(A–D)** and kidney **(E–H)** collected at 14 dpc: **(A, E)** naïve baseline; **(B, F)** single-dose rNDV-IBV-T/B; **(C, G)** booster-dose rNDV-IBV-T/B; and **(D, H)** sham-vaccinated challenge control. Compared with the challenge control, both vaccinated groups showed qualitatively milder tracheal and renal lesions. The booster-dose group exhibited largely preserved tracheal epithelial continuity and no severe renal congestion or widespread structural disruption. Histopathological findings were evaluated descriptively; no semiquantitative lesion scoring or inferential statistical comparison was performed. Scale bars = 100 μm.

Renal histopathology followed a similar qualitative pattern. The naïve baseline group (**Figure 4E**) showed normal tubular architecture, with regularly arranged epithelial cells and no evident interstitial inflammation. Both vaccinated groups showed qualitatively milder renal lesions than the sham-vaccinated challenge control. The representative booster-dose section (**Figure 4G**) showed largely preserved tubular architecture with only mild residual tubular changes. In contrast, the sham-vaccinated challenge control (**Figure 4H**) exhibited extensive tubular epithelial degeneration and necrosis, tubular dilation, proteinaceous casts, and interstitial inflammatory-cell infiltration. Because histopathology was evaluated descriptively without a semiquantitative lesion-scoring system, these observations should not be interpreted as statistically significant differences between the vaccination regimens. No significant lesions were observed in the lung or bursa of Fabricius in any group, consistent with the respiratory and renal tropism of QX-like IBV.

### 3.7 Viral shedding after challenge

Quantitative PCR analysis revealed distinct viral RNA shedding kinetics among the groups (**Figure 5**). In oropharyngeal swabs, the sham-vaccinated challenge control group reached its highest mean viral RNA load at 4 dpc (6.58 ± 0.15 log_10_ copies/swab), compared with 5.71 ± 0.36 and 5.41 ± 0.10 log_10_ copies/swab in the single-dose and booster-dose groups, respectively. Compared with the challenge control, the booster-dose group had significantly lower oropharyngeal viral RNA loads at 2, 4, and 8 dpc, whereas the single-dose group had significantly lower loads at 4, 6, and 8 dpc. No significant difference between the booster-dose and challenge-control groups was detected at 6, 10, 12, or 14 dpc. The booster-dose group also had significantly lower oropharyngeal viral RNA loads than the single-dose group at 2 and 12 dpc; no other between-vaccine differences were significant.

**Figure 5:**
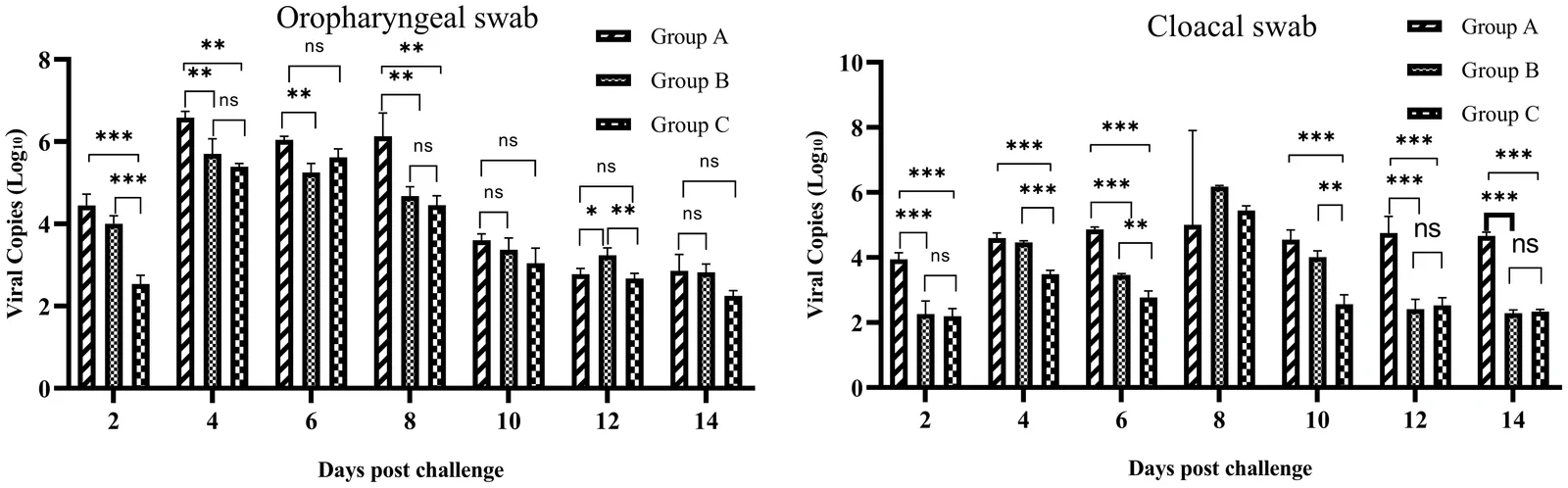
Serial cross-sectional assessment of IBV RNA loads in oropharyngeal and cloacal swabs following QX-type challenge. Oropharyngeal (left panel) and cloacal (right panel) IBV RNA loads were quantified by qRT-PCR at the indicated days post-challenge. At each time point, three chickens per group were sampled from the 10-bird terminal cohort. Each swab was tested in technical triplicate, and the technical replicates were averaged to obtain one bird-level value. Data are presented as mean ± SD (n=3 chickens per group per time point). Group differences were evaluated separately at each time point using one-way ANOVA followed by Tukey’s multiple-comparison test. One cloacal sample from Group A at 8 dpc showed detectable amplification below the LLOQ and was retained as a left-censored observation by assigning one-half of the LLOQ on the original copy-number scale before log_10_ transformation. No significant differences among the three groups were detected at this time point. *p<0.05, **p<0.01, and ***p<0.001; ns, not significant.

In cloacal swabs, the booster-dose group had significantly lower viral RNA loads than the sham-vaccinated challenge control at 2, 4, 6, 10, 12, and 14 dpc, but not at 8 dpc. The single-dose group had significantly lower loads than the challenge control at 2, 6, 12, and 14 dpc, but not at 4, 8, or 10 dpc. The booster-dose group also had significantly lower loads than the single-dose group at 4, 6, and 10 dpc. At 8 dpc, one value in the sham-vaccinated challenge control group was below the LLOQ and was assigned one-half of the LLOQ before log_10_ transformation; after inclusion of this value, no significant differences were detected among the three groups. Because qRT-PCR detects viral RNA rather than infectious virus, these findings do not establish a corresponding reduction in infectious-virus shedding or transmission.

## 4 Discussion

The persistent emergence of QX-like (GI-19) IBV variants, characterized by S1 <80% nucleotide identity to Mass-type M41 and frequent recombination with vaccine strains (4/91, H120, QXL87), continues to undermine conventional vaccination in China and Europe (12). While QXL87 (GI-19 live attenuated) is deployed domestically, recent QX field isolates exhibit R-values <65% against QXL87 and novel cleavage motifs (PRRRR substituting classical HRRRR) absent from prototype QX, indicating ongoing antigenic drift even within GI-19 (32). In this context, our study extends the rNDV-IBV-T/B candidate—previously validated against M41 (27)—to a QX-like challenge, and demonstrates that a multi-epitope strategy focusing on conserved S1 B-cell hotspots plus QX-derived BF2-restricted CTL epitopes (P8, P9) overcomes the M41–QX divergence to confer solid protection. Notably, the lack of QX-associated mortality in the present study—despite severe histopathological lesions and high viral loads in controls—aligns with the age-dependent pathogenicity of GI-19 strains, which exhibit markedly reduced lethality in chickens beyond 28 days of age (31). This contrasts with our prior M41 trial (27), where challenge at 21 days of age resulted in sporadic deaths, underscoring how host maturity modulates the clinical outcome of IBV infections even within the same vaccine vector framework. A major immunological observation of this study was that improved protection after booster immunization occurred in parallel with an increased frequency of splenic CD3^+^CD8^+^ T cells, which coincided with reduced pathology and shedding. However, because immune and protection endpoints were measured in different birds and no functional depletion or adoptive-transfer experiments were performed, these data do not establish a causal role for CD8^+^ T cells or demonstrate that cellular immunity contributes more than neutralizing antibodies.

A pivotal distinction between our construct and other NDV-LaSota-vectored IBV multi-epitope vaccines lies in epitope cassette composition rather than vector backbone—both rNDV-IBV-T/B (this study and our previous work (27)) and rLa Sota/SBNT (28) use the NDV-LaSota strain, but differ in which T-cell epitopes are paired with the conserved S1 B-hotspots. Qin et al. paired the three S1 B-hotspots with four N-protein T-cell epitopes (N_211-230_, N_261-280_ dual-specific, N_271-290_, N_381-400_), protecting against QX-SD and TW-GD but failing against M41—attributed to the N-CTL repertoire being insufficient when B-cell conservation alone could not bridge the M41–QX serotype gap. In contrast, rNDV-IBV-T/B pairs the same S1 B-cell hotspots with S1-derived BF2-restricted CTL epitopes (P8 and P9 from a QX-like strain). We found that the booster regimen produced the most consistent reduction in cloacal viral RNA, with significantly lower loads than the sham-vaccinated challenge control at 2, 4, 6, 10, 12, and 14 dpc. No significant group difference was detected at 8 dpc because of substantial inter-animal variability. These findings support an effect of booster immunization on viral RNA shedding but should not be interpreted as evidence of reduced infectious-virus shedding or fecal–oral transmission. Several rNDV constructs expressing full-length IBV S1 have also been reported to reduce viral RNA shedding (22, 23, 33). This observation suggests that, within the same NDV-LaSota backbone, pairing S1 B-hotspots with S1-native CTL may offer a different protective profile compared to N-CTL, a hypothesis that warrants direct experimental comparison. Critically, this design flexibility derives entirely from the modularity of the NDV-LaSota backbone, which can be readily reconfigured with diverse epitope cassettes targeting evolving viral lineages. Although the LaSota backbone has a well-characterized safety and immunogenicity record, whether insertion of this specific multi-epitope cassette preserves these properties requires direct evaluation.

Our immunological data shed light on this protection gradient. Booster immunization increased the proportion of CD3^+^CD8^+^ splenocytes to 41.3% ± 0.6%, compared with 31.9% ± 0.9% in the sham-vaccinated challenge control group, whereas CD3^+^CD4^+^ T-cell proportions remained similar among groups. This aligns with classical IBV pathogenesis studies showing that tracheal CTL infiltrates correlate with early viral clearance, whereas neutralizing antibodies dominate later phases (34–36). The peak log_2_ GMT was 5.80 (95% CI, 5.24–6.36), numerically approximately 1.0 log_2_ lower than that reported in the previous M41 study (27). Because these measurements were obtained in separate experiments, this descriptive comparison should not be interpreted as evidence of relative immunogenicity or efficacy. Historical studies likewise support an association between vaccine-induced humoral immunity and protection against IBV challenge, although neutralizing antibody titers are not directly comparable across different assays and challenge models (31, 37). Neutralizing antibody responses and increased splenic CD3^+^CD8^+^ T-cell frequencies were observed in parallel with improved protection. However, because these immune and protection endpoints were measured in different chickens, and no individual-level correlation or functional experiment was performed, their relative contributions cannot be resolved from the present study. The increased CD3^+^CD8^+^ T-cell frequency should therefore be interpreted as a group-level immunological association rather than evidence that CTL responses represent the dominant protective mechanism. This distinction is particularly informative when comparing our construct to the N-CTL-based rLaSota/SBNT construct, which induced comparable VN titers against QX yet failed to protect against M41 (28). Although the two constructs differed in their reported outcomes across challenge models, these non-concurrent experiments cannot establish that their different T-cell epitope repertoires caused the observed differences. NDV-protective efficacy of rNDV-IBV-T/B was previously validated and thus not reassessed here.

The booster regimen produced the most consistent reduction in cloacal viral RNA loads. Whether this reduction translates into decreased fecal–oral transmission remains to be determined by virus isolation, infectivity titration, and direct transmission studies. The present findings support further evaluation of a prime–boost regimen at 3 and 24 days of age in breeders or long-lived layers; however, its superiority to a single-dose regimen under commercial conditions requires direct field testing.

Several limitations warrant emphasis. First, experiments were in SPF chickens devoid of maternal antibody. In commercial flocks, maternally derived NDV antibodies can neutralize the rNDV-LaSota vector, potentially dampening IBV epitope priming (3); this needs evaluation in commercial-cross broilers with defined NDV-MDA levels. Second, QX diversity is increasing: recent Chinese isolates carry an H536P substitution creating a PRRRR cleavage motif (vs. classical HRRRR) and recombine with 4/91/H120/QXL87 (32), while GVI-1 lineages in southern China evade H120+LaSota programs (12). Whether P8/P9 CTL epitopes and the S1 B-cell hotspots remain conserved across these recombinants warrants epitope-mapping validation. Conserved S2-derived B-cell targets, including the HR2 epitopes ^¹⁰⁴⁰^KWWND^¹⁰⁴⁴^ and ^¹⁰⁴⁶^KHELPDF^¹⁰⁵²^ (29), may complement S1-hotspot designs; S2-derived peptides have also shown broad antibody reactivity in diagnostic applications (38). Third, the long-term genetic stability of the multi-epitope insert over >10 in vivo passages, and any recombination potential between rNDV-IBV-T/B and field NDV/IBV, remain to be defined prior to field trials.

Several limitations of this study warrant acknowledgment. First, sample sizes for flow cytometry (n=3/group), viral RNA analysis (n=3/group/time point), histopathology (n=3/group), and ciliostasis (n=2–3/group/time point) were modest, which reduces statistical power to detect smaller effect sizes and limits the precision of estimated group means. The findings, particularly the CD8^+^ T-cell data, should be interpreted with this in mind. Second, the central mechanistic inference—that CD8^+^ T-cell expansion contributes to protection—is based on associative group-level trends rather than causal experiments (e.g., CD8^+^ depletion or adoptive transfer). We have therefore used descriptive language such as “associated with” throughout the manuscript. Third, comparative claims against historical studies (27, 28) rely on non-concurrent data confounded by differences in virus strain, dose, and animal batch; these comparisons should be considered preliminary pending head-to-head validation. Fourth, no significant difference among the three groups was detected in cloacal viral RNA loads at 8 dpc because of substantial inter-animal variability, including one left-censored value in the sham-vaccinated challenge control group. Furthermore, viral shedding was quantified solely by qRT-PCR, which measures RNA copies rather than infectious virus titer; the true impact on transmission risk remains to be confirmed by virus isolation.

In conclusion, rNDV-IBV-T/B, previously evaluated against M41, also conferred protection against QX-like IBV in the present SPF-chicken model. The booster regimen was associated with increased splenic CD3^+^CD8^+^ T-cell frequencies and a more consistent reduction in cloacal viral RNA loads. These findings support further evaluation in chickens with maternally derived antibodies, against additional IBV genotypes, and under field-relevant safety and potency conditions.

## Data Availability

The original contributions presented in the study are included in the article/**Supplementary Material**. Further inquiries can be directed to the corresponding authors.
