# Supplementary material for: A recombinant Newcastle disease virus-vectored multi-epitope vaccine confers protection against QX-like infectious bronchitis virus in chickens
Source: Front Immunol. 2026 Sep 22;17:1949497. doi: 10.3389/fimmu.2026.1949497 (PMC13640013; doi:10.3389/fimmu.2026.1949497)
Supplement: Supplementary file 1 [file Table1.docx]

**Supplementary Table S1: Detailing the animal allocation**

| Time Point / Assay | Number of chickens | Relationship to total / use |
| --- | --- | --- |
| Initial allocation | 20 | Three experimental groups; total n=60 |
| Pre-challenge flow cytometry | 3 | Terminal spleen collection; 17 chickens remained for challenge |
| Challenge cohort | 17 | Chickens entering the QX challenge |
| Ciliostasis, 5 dpc | 2 | Terminal; independent chickens |
| Ciliostasis, 7 dpc | 2 | Terminal; independent chickens |
| Ciliostasis, 9 dpc | 3 | Terminal; independent chickens |
| 14-dpc terminal cohort | 10/group | Chickens remaining after the terminal ciliostasis collections |
| Viral RNA sampling | 3/group/time point | Time-specific random subsets selected from the 10-chicken terminal cohort; non-terminal swabbing; the same chickens were not necessarily sampled repeatedly |
| Histopathology | 3/10 per group | Independently selected from the 10-chicken terminal cohort at 14 dpc |
| VN serum sampling | 5/time point | Non-terminal sampling |
| Naïve baseline group | 5 | Unvaccinated and unchallenged |

The total of 20 chickens per group was distributed as above. An additional 5 naïve chickens served as unchallenged baseline controls for histopathology.
